# Dietary Fermented Soy Extract and Oligo-Lactic Acid Alleviate Chronic Kidney Disease in Mice via Inhibition of Inflammation and Modulation of Gut Microbiota

**DOI:** 10.3390/nu12082376

**Published:** 2020-08-08

**Authors:** Li-Xia He, Hamid M. Abdolmaleky, Sheng Yin, Yihong Wang, Jin-Rong Zhou

**Affiliations:** 1 Nutrition/Metabolism Laboratory, Department of Surgery, Beth Israel Deaconess Medical Center, Harvard Medical School, Boston, MA 02215, USA; Lixia_He@dfci.harvard.edu (L.-X.H.); sabdolma@bidmc.harvard.edu (H.M.A.); syin@bidmc.harvard.edu (S.Y.); 2 Feihe Nutrition Laboratory, Department of Surgery, Beth Israel Deaconess Medical Center, Harvard Medical School, Boston, MA 02215, USA; 3 Department of Pathology and Laboratory of Medicine, Warren Alpert Medical School of Brown University, Providence, RI 02912, USA; Yihong_Wang@brown.edu

**Keywords:** chronic kidney disease, inflammation, fermented soybean, oligo-lactic acid, gut microbiota, stem cells

## Abstract

Chronic kidney disease (CKD) is a global epidemic with an increasing prevalence worldwide. Effective preventive strategies are urgently needed. This study aimed to investigate the effect of nutraceutical components, a fermented soybean product (ImmuBalance, IMB) and an oligo-lactic acid product (LAP), on the prevention of adenine-induced CKD in mice. Female C57BL/6 mice were randomly assigned into following experimental groups: negative control; model control; and models treated with IMB at 250 or 1000 mg/kg body weight (BW), LAP at 1000 or 2000 mg/kg BW, and IMB/LAP combinations. The CKD model was established by intraperitoneal injection of adenine daily for 4 weeks, and treatments started 2 weeks before adenine injection and ended after 10 weeks. Compared with the model control, the treatments did not significantly alter the body weight or food intake. Both IMB and LAP, especially their combination, significantly inhibited tubular dilation, tubulointerstitial degeneration or atrophy, interstitial chronic inflammation and acute inflammation in the kidneys of CKD mice, and significantly decreased serum cystatin C levels. IMB or LAP significantly reversed CKD-associated increases of circulating and kidney levels of inflammatory cytokines, circulating levels of kidney injury biomarkers, and kidney levels of stem cell biomarkers, and significantly reversed CKD-associated reduction of cecum *Clostridium leptum* group. Our results suggest that dietary supplementation of IMB or LAP may significantly delay the development and/or progression of CKD.

## 1. Introduction

Chronic kidney disease (CKD) is a slow and progressive loss of kidney function, and poses serious health problems. It is estimated that about 15% of the U.S. population, or 37 million U.S. adults, would have had CKD in 2019 [1]. CKD is an important contributor to morbidity and mortality from noncommunicable diseases, and this disease should be actively addressed to meet the United Nation’s (UN) Sustainable Development Goal target to reduce premature mortality from non-communicable diseases by one-third by 2030 [2]. CKD is a syndrome with different etiologies. While inflammation is one of core pathological features of CKD, research data indicate that CKD itself “is recognized as a proinflammatory state” [3] and uremic environment induces inflammation [4] in diverse tissues, generating other diseases, mainly cardiovascular diseases. There are many factors that lead to the setting of the inflammatory status in CKD, including increased production of proinflammatory cytokines and oxidative stress, chronic and recurrent infections, altered metabolism of adipose tissue, and gut microbiota dysbiosis, an underestimated source of microinflammation [5].

Although genetic factors play a role in the incidence and progression of CKD, it is recognized that nutritional factors play an essential role in the cause, progression, and prevention of the disease. It is estimated that more than 24% of CKD cases in industrialized countries can be attributed to nutritional factors [6]. More than 70% of kidney failure cases are associated with diabetes and hypertension, the major nutrition-related metabolic diseases. Although the treatment of diabetes and hypertension can postpone the development of CKD, a large fraction of affected patients proceed to CKD despite medical interventions. More effective dietary and nutritional approaches are urgently needed to prevention the development and progression of CKD.

The potential renal benefits of soybean products have considerable public health significance because of the increasing worldwide prevalence of renal disease [7]. The previous research evidence showed that replacing animal protein with soy protein contributed to a reduction in hyperfiltration and glomerular hypertension, with resultant protection from diabetic nephropathy [8,9]. Nevertheless, a meta-analysis of 12 clinical studies involving 280 patients with chronic renal disease found that dietary soy protein did not affect the glomerular filtration rate, although it significantly decreased serum creatinine, serum phosphorus, inflammation, and proteinuria in predialysis patients [10].

It has been demonstrated that lactic acid plays important roles in multiple conditions of health and disease, including energy regulation, anti-inflammation, immune modulation, wound repair, and cancer progression [11]. However, very few studies have reached its molecule fundamentals and associated pathways. Further investigation and a better understanding of lactic acid will be beneficial for medical practice and benefit patient care.

Our overall hypothesis is that dietary or nutritional interventions that inhibit inflammation may provide an effective strategy for the prevention and/or delay the progression of CKD. In this study, we proposed to examine the effects of two novel nutritional components, a specially prepared soybean fermentation product ImmuBalance (IMB) and a unique oligo-lactic acid product (LAP), on inhibiting inflammation and alleviating inflammation-induced kidney damage in a mouse model of adenine-induced CKD [12,13]. We also studied the quantity of common bacteria in mice cecum to examine potential influences of our nutritional interventions in gut microbiome and the prevention of CKD.

## 2. Materials and Methods

### 2.1. Materials

A soy extract, IMB, was prepared by a koji fermentation of defatted soybeans with *Aspergillu soryzae* and lactic acid bacteria (*Pediococcus parvulus* and *Enterococcus faecium*) according to a proprietary fermentation technology, followed by water extraction and purification of Koji polysaccharides^®^. Hydrolysis analysis showed that this polysaccharide mainly consisted of arabinose (41.4%), galactose (23.7%), and xylose (10.4%). IMB was provided by Nichimo Biotics Co., Ltd., Tokyo, Japan. Oligo-lactic acid product (LAP) was a condensate of about nine ester-linked molecules of L-lactic acid that was purified from fermentation products of sugar beet and corn with *Lactobacilli* according to a proprietary process. LAP was provided by LifeTrade Co., Ltd., Tokyo, Japan.

### 2.2. Animal Study

Female C57BL/6 mice (6–8 weeks of age) were purchased from Taconic (Germantown, NY, USA). After one week of acclimation, mice consuming the AIN-93M diet were randomly assigned into one of the following experimental groups (*n* = 8/group): (1) negative control (NC); (2) CKD model control (MC); (3–4) CKD models with oral gavage of IMB at 250 (IMB-L) and 1000 mg/kg BW (IMB-H), respectively; (5–6) CKD models with oral gavage of LAP at 1000 (LAP-L) and 2000 mg/kg BW (LAP-H), respectively; and (7–8) CKD models with oral gavage of IMB-L/LAP-L combination and IMB-H/LAP-H combination, respectively. The CKD model was established by intraperitoneal injection of adenine at 100 mg/kg BW daily for 4 weeks. The treatments started 2 weeks before adenine injection to evaluate the preventive effect, and ended 4 weeks after the last adenine injection. The animal experiment was performed in accordance with National Institutes of Health guidelines and approved by the Institutional Animal Care and Use Committee of Beth Israel Deaconess Medical Center (approval number: 028-2018).

Food intake and body weight were measured weekly. At the end of the experiment, the mice were sacrificed, blood samples were collected, and the resulting serum samples were saved at −80 °C for further analysis. One kidney (left) was dissected and fixed in 10% buffer-neutralized formalin, paraffin-embedded, and sectioned at 4 μm thickness for histopathology and immunohistochemistry analyses. Another kidney and cecum containing feces were harvested, snap-frozen in liquid nitrogen, and then saved at −80 °C for further analysis.

### 2.3. Histopathology Analysis

Kidney tissue slides were processed with hematoxylin and eosin (H & E) stain, periodic acid-Schiff (PAS), and Masson’s trichrome stain for histopathological evaluation by scoring for tubular dilation, tubulointerstitium degeneration, interstitial chronic inflammation, and acute inflammation. For the acute process, interstitial acute inflammation and tubular dilation without tubular epithelial thinning or basement membranes thickening were evaluated. For the chronic process, tubulo-interstitial degeneration including tubular atrophy and interstitial fibrosis, as well as chronic inflammation were evaluated. Features of tubular epithelial thinning and basement membrane thickening were evaluated with reviewing the PAS special stains. A semi-quantitative score system was implemented. A score of 0–3, denoting increasingly severe abnormality, was assigned by a pathologist blinded to the experimental design and identity of the samples. In each mouse tissue, the tubular dilation was graded as 0 (no pathology), 1 (<25%), 2 (25–50%), or 3 (>50%); the tubular atrophy and fibrosis was graded as 0 (no pathology), 1 (focal, small patchy), 2 (cortical/circumferential), or 3 (global); the interstitial chronic inflammation was graded as 0 (none), 1 (scant 1–2 foci, mild), 2 (2–4 foci, mild), or 3 (>5 foci, dense); and the acute inflammation was graded as 0 (none), 1 (focal), 2 (patchy), or 3 (diffused).

### 2.4. Multiplex Sandwich Immunoassays for Measurement of Blood Levels of Cytokines and Kidney Toxicity Biomarkers

Multiplex sandwich immunoassays were performed in Luminax Magpix System (Millipore Sigma, Burlington, MA, USA) to determine the levels of cytokines (ProcartaPlex Mouse 11 Cytokine Premixed Kit, ThermoFisher, Carlsbad, CA, USA) and kidney toxicity biomarkers (Milliplex Mouse 3 and 5-KidneyInjuryPremixed Kits, Millipore, Billerica, MA, USA) in serum samples following the established protocols in accordance with the manufacturer’s instructions. The 11 cytokines panel included interferon (IFN)-γ, interleukin (IL)-1α, IL-1β, IL-6, IL-10, IL-12p70, IFN-γ-inducible protein 10 (IP-10, CXCL10), keratinocyte-derived chemokine (KC), monocyte chemotactic protein-1 (MCP-1), tumor necrosis factor (TNF)-α, and vascular endothelial growth factor A (VEGF-A). The three kidney injury factors comprised kidney injury molecule-1 (KIM-1), renin, and tissue inhibitor of metalloproteinases 1 (TIMP-1); and the five kidney injury factors comprised clusterin, cystatin C, epidermal growth factor (EGF), lipocalin-2 (LCN2)/neutrophil gelatinase-associated lipocalin (NGAL), and osteopontin (OPN).

A five-parameter model was used to calculate final concentrations and values were expressed in pg/mL. Final analyses of Luminex data for cytokines took consideration of data that fell within the detection limits of the Luminex assay. Concentrations obtained below the sensitivity limit of detection (LOD) of the method were calculated as LOD/2 for statistical comparisons.

### 2.5. Quantitative Polymerase Chain Reaction (qPCR) for Determination of Gut Microbiota

The mouse cecum was collected and microbial genomic DNA was extracted from 200 mg of cecal sample using the E.Z.N.A. ^®^Stool DNA Kit (D4015, Omega Bio-Tek, Inc., Norcross, GA, USA)/QIAamp DNA Stool mini kit (Qiagen) according to the manufacturer’s instructions. Total DNA was quantified and its purity was assessed using NanoDrop™ 2000 spectrophotometers (ThermoFisher, Carlsbad, CA, USA). Nuclease-free water was used as a blank.

The amount of total microbiota was estimated using the universal primers, Uni331F and Uni797R, which amplified a conserved region of the 16S rRNA for most of the common microbiota [14]. The following representative dominant/subdominant groups from four major phyla of gut microbiota were chosen: *Atopobium* cluster, *Bifidobacterium* genus, *Bacteroides fragilis* group, *Clostridium coccoides* group, *Clostridium perfringens* group, *Desulfovibrio* genus, *Enterobacteriaceae* family, *Lactobacillus* genus, *Clostridium leptum* group, and *Prevotella* genus. qPCR assays were performed using SYBR Green qPCR Master Mix on a CFX384 Touch™ Real-Time PCR Detection System (BioRad Laboratories, Hercules, CA, USA). The amplification protocol consisted of 1 cycle of 95 °C for 20 s, followed by 40 cycles of 95 °C for 15 s, appropriate annealing temperature for 30 s and 72 °C for 35 s, and finally 1 cycle of 60–94 °C with 0.5 °C increments, 15 s dwell time. The results were normalized to 16S ribosomal (universal) DNA sequences and expressed as the relative difference using the 2^ΔΔCt^ method.

### 2.6. Quantitative Real-Time PCR (qRT-PCR) for Analysis of Inflammation- and Stem Cells-Related Genes in Kidney Tissues

Quantitative analysis of inflammatory cytokine (IL-1, IL-6, TNF-α, F4/80, transforming growth factor (TGF)-β1, MCP-1, toll-like receptor (TLR)-4, and IL-1β) and stem cell-related biomarkers (CD133, Pax-2, Six2, CD11β, Oct-4, CD29, CD44, NANOG, vimentin, Wnt-4, WT-1, and nestin) was performed by qRT-PCR. Total RNA was isolated from kidney samples and reverse transcribed into cDNA using iScript Reverse Transcription Supermix for PCR. Synthesized cDNA was subjected to qRT-PCR assay with specific primers (Table 1) and ScoAdvanced Universal SYBR SuperMix (BioRad). The thermal cycling conditions used were as follows: 1 cycle of 95 °C for 20 s, followed by 40 cycles of 95 °C for 15 s, appropriate annealing temperature for 30 s and 72 °C for 35 s, and finally 1 cycle of 60–94 °C with 0.5 °C increments, 15s dwell time. All mRNA quantification data were calculated using the 2^ΔΔCt^ method and normalized to glyceraldehyde 3-phosphate dehydrogenase (GAPDH), presented as fold changes compared with the controls.

### 2.7. Statistical Analysis

Data were expressed as the group mean ± standard deviation and analyzed by one-way analysis of variance (ANOVA) test, followed by multiple comparison of least-significant difference (equal variances assumed) or Dunnett’s T3-test (equal variances not assumed) to evaluate the difference of parametric samples among groups. When raw data or log-transformed data did not meet the statistical criteria for the assumption of normality showing equal variance, the nonparametric Kruskal–Wallis or Mann–Whitney test was used to determine statistical differences, and Bonferroni correction was used to correct the *p*-value. Differences were considered to be statistically significant at *p* < 0.05. A Spearman correlation analysis was used to investigate the relationship between histopathology and biomarkers. All analyses were carried out using IBM SPSS Statistics version 20.0 and GraphPad Prism 5 Software.

## 3. Results

### 3.1. Effects of IMB and LAP Treatments on Body Weight

**Figure 1:**
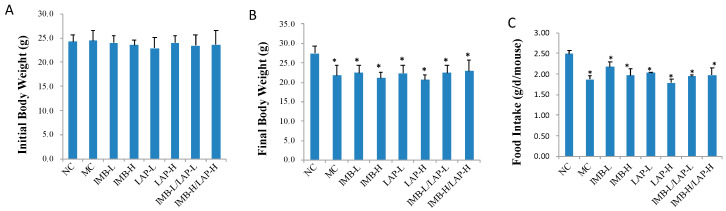
Effects of IMB and LAP treatments on body weight of CKD mice. (**A**), initial body weight; (**B**), final body weight; (**C**), food intake. Data was presented as Mean ± SD, and analyzed by ANOVA. Within the panel, values with a superscript * are significantly different from that of the NC, *p* < 0.05.

There was no significant difference in initial body weight among all groups (*p* > 0.05) (Figure 1A). Induction of CKD significantly reduced final body weights and food intake in all model groups (*p* < 0.05) (Figure 1B,C). Compared with the model control, dietary treatments did not significantly alter final body weights (Figure 1B) or food intake (Figure 1C) (*p* > 0.05).

### 3.2. Effects of IMB and LAP Treatments on Kidney Inflammation and Damage

**Figure 2:**
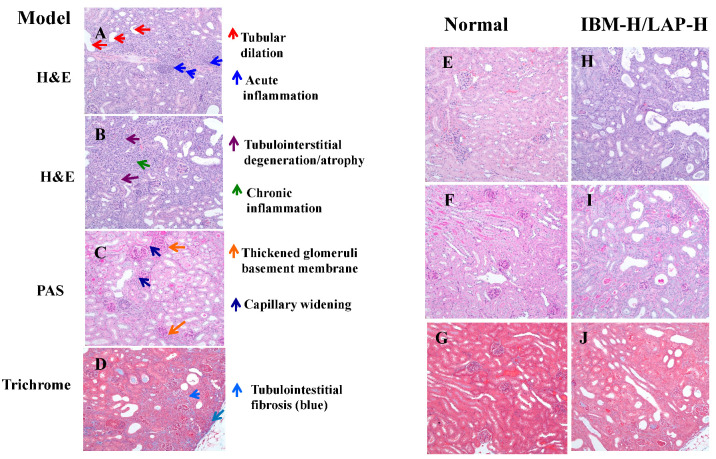
Histopathological evaluation of kidney lesions. Representative histopathology slides from the adenine-induced CKD mice showing tubular dilation and acute inflammation (**A**), tubulointerstitial degeneration/atrophy and chronic inflammation (**B**), thickened glomeruli basement membrane and capillary widening (**C**), and tubulointerstitial fibrosis (**D**). The representative histology slides from the normal mice showing no lesions (**E**–**G**), and the representative histology slides from the effective treatment mice showing reduced lesions (**H**–**J**).

The effects of treatments on kidney inflammation and damage were evaluated histopathologically by H & E, PAS, and trichrome staining. The statistical analysis results of histopathological scores of kidney tissues are presented in Table 2, and the representative results are shown in Figure 2. Tubular dilation without basement membrane thickening was considered as an acute process and was used to evaluate the acute change. Tubular dilation along with other features were evaluated under the tubular atrophy. Tubular atrophy, interstitial fibrosis, and chronic inflammation were evaluated as chronic changes. The kidney tissues in the adenine-treated mice showed tubular dilation (Figure 2A), acute inflammation (Figure 2A), tubulointerstitial degradation (Figure 2B), interstitial chronic inflammation (Figure 2B), capillary widening (Figure 2C), thickened glomeruli basement membrane (Figure 2C), and tubulointerstitial fibrosis (Figure 2D), whereas the kidney tissues from normal control mice did not show histopathological lesions (Figure 2E–G). These histopathological parameters were improved by IMB and/or LAP treatments (Figure 2H–J, images were from one mouse treated with the IMB-H/LAP-H combination group).

Compared with the NC, the MC showed significantly higher levels of tubular dilation, tubulointerstitial degeneration/atrophy, chronic inflammation, and acute inflammation (*p* < 0.05 in all histopathological lesions). The administration of LAP-L significantly alleviated the kidney inflammation and damage, and inhibited tubular dilation (*p* < 0.05), tubulointerstitial degeneration/atrophy (*p* < 0.05), and chronic inflammation (*p* < 0.05), and IMB-H significantly inhibited tubulointerstitial degeneration/atrophy (*p* < 0.05). In particular, the IMB-H/LAP-H combination significantly inhibited all measured histopathological parameters (*p* < 0.05), and the IMB-L/LAP-L combination significantly alleviated tubular dilation and interstitial chronic inflammation (*p* < 0.05). These results suggest that the combination of IMB and LAP, especially at the high doses, may further enhance the protective effect on kidney damage/inflammation, although the apparent combination effect (additive or synergistic) was not obvious. While IMB and LAP treatment alone showed some beneficial effects, no clear dose-dependent effect was determined.

### 3.3. Effects of IMB and LAP Treatments on Circulating Levels of Cytokines and Kidney Injury Biomarkers

**Figure 3:**
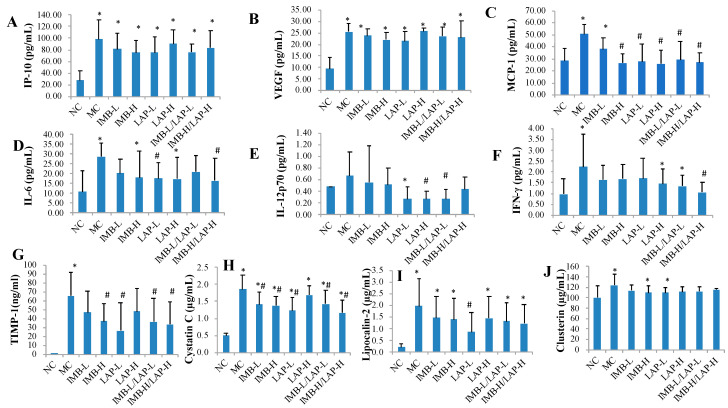
Effects of IMB and LAP supplements on circulating levels of cytokines and kidney injury biomarkers. The circulating cytokines levels of IP-10 (**A**), VEGF (**B**), MCP-1 (**C**), IL-6 (**D**), IL-12p70 (**E**) and IFN-γ (**F**); the circulating levels of kidney injury markers TIMP-1 (**G**), cystatin (**H**), lipocalin-2 (**I**) and clusterin (**J**). Values are expressed as Mean ± SD, n = 8. Within each panel, values with the superscript symbols “*” and “#” are significantly different from the NC and MC, respectively, *p* at least <0.05.

Figure 3 (Figure 3A–F) shows that the serum levels of cytokines IP-10, VEGF, MCP-1, IL-6, and IFN-γ in the MC were significantly increased compared with the NC (*p* < 0.05), whereas the serum level of IL-12p70 in the MC was non-significantly increased (*p* > 0.05) (Figure 3E). Compared with that of the MC, MCP-1 levels were significantly reduced in all treatment groups except the IMB-L group (*p* < 0.05, Figure 3C), IL-6 levels were significantly reduced in the LAP-L and the IMB-H/LAP-H groups (*p* < 0.05, Figure 3D), IL-12p70 levels were significantly reduced in the LAP-H and the IMB-L/LAP-L groups (*p* < 0.05, Figure 3E), and IFN-γ level was significantly reduced in the IMB-H/LAP-H group (*p* < 0.05, Figure 3F). 

Compared with that of the NC, the serum levels of kidney injury biomarkers TIMP-1, cystatin C, lipocalin-2, and clusterin in the MC were significantly increased (*p* < 0.05, Figure 3G–J, respectively). Compared with that of the MC, cystatin C levels were significantly decreased in all treatment groups except the LAP-H group (*p* < 0.05, Figure 3H), TIMP-1 levels were significantly reduced in the IMB-H, LAP-L, IMB-L/LAP-L, and IMB-H/LAP-H groups (*p* < 0.05, Figure 3G), and lipocalin-2 levels were significantly reduced in the LAP-L group (*p* < 0.05, Figure 3I). On the other hand, clusterin levels were not significantly altered by the treatments (Figure 3J).

### 3.4. Effects of IMB and LAP Treatments on the Expression Levels of Inflammatory Cytokines in Kidney

**Figure 4:**
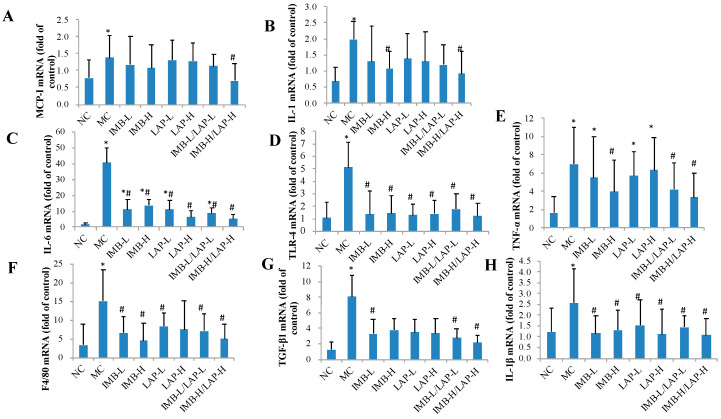
Effects of IMB and LAP supplements on the gene expression levels of inflammatory cytokines in kidney. MCP-1 (**A**), IL-1 (**B**), IL-6 (**C**), TLR-4 (**D**), TNF-α (**E**), F4/80 (**F**), TGF- β 1 (**G**), and IL-1 β (**H**). Values are expressed as Mean±SD, n=8. Within each panel, values with the superscript symbols “*” and “#” are significantly different from the NC and MC, respectively, *p* at least <0.05.

As shown in Figure 4, when compared with the NC group, the MC group showed significantly increased expression levels of MCP-1, IL-1, IL-6, TLR-4, TNF-α, F4/80, TGF-β1, and IL-1β genes in kidney (*p* < 0.05). When compared with the MC, the experimental treatments of IMB or LAP significantly reduced the expression levels of IL-1 (Figure 4B, except IMB-L, LAP-L, LAP-H, and IMB-L/LAP-L), IL-6 (Figure 4C), TLR-4 (Figure 4D), TNF-α (Figure 4E, except IMB-L, LAP-L, and LAP-H), F4/80 (Figure 4F, except LAP-H), TGF-β1 (Figure 4G, except IMB-H, LAP-L, and LAP-H), and IL-1β (Figure 4H). The IMB-H/LAP-H treatment also reduced the expression level of MCP-1 (Figure 4A). It is also important to note that the IMB or LAP treatments reduced adenine-induced expression of MCP-1, IL-1, TLR-4, F4/80, TGF-β1, and IL-1β genes in kidney to the levels that were comparable to the NC (*p* > 0.05). These results further supported that IBM or LAP could significantly reduce kidney inflammation levels in kidney.

### 3.5. Effects of IMB and LAP Treatments on the Expression Levels of Stem Cell-Related Genes in Kidney

**Figure 5:**
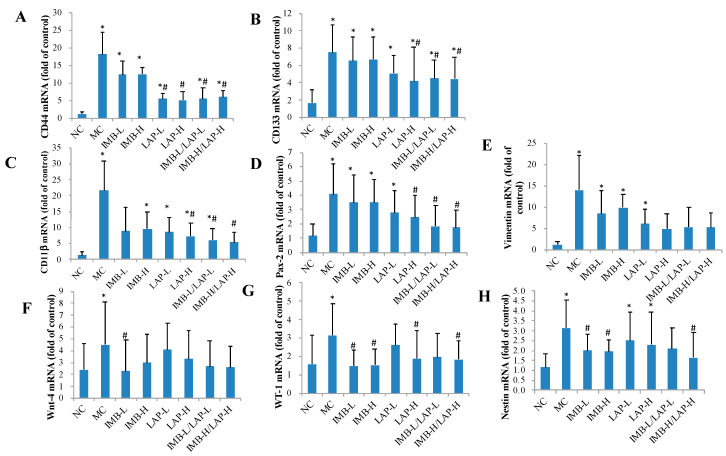
Effects of IMB and LAP on the gene expression levels of stem cells markers in kidney. CD44 (**A**), CD133 (**B**), CD11β (**C**), Pax-2 (**D**), vimentin (**E**), Wnt-4 (**F**), WT-1 (**G**), and nestin (**H**). Values are expressed as Mean±SD, n=8. Within each panel, values with the superscript symbols “*” and “#” are significantly different from the NC and MC, respectively, *p* at least <0.05.

Compared with the NC, the MC showed significantly elevated CD44, CD133, CD11β, Pax-2, vimentin, Wnt-4, Wt-1, and nestin mRNA levels in kidney (Figure 5, *p* < 0.05). The treatments with LAP-H and the two combinations ameliorated the imbalance changes of CD44, CD133, CD11β, and Pax-2 mRNA levels compared with the positive control (*p* < 0.05), and the treatments with IMB (low or high dose) and the combination of IMB-H/LAP-H reduced Wt-1 and nestin mRNA levels (*p* < 0.05).

### 3.6. Effects of IMB and LAP Treatments on Gut Microbiota

**Figure 6:**
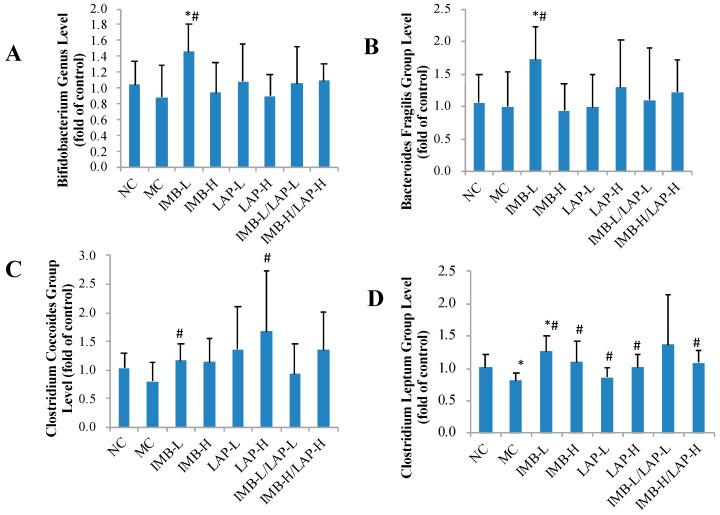
Effects of IMB and LAP supplements on gut microbiota levels. Bifidobacterium genus (**A**), Bacteroides fragilis group (**B**), Clostridium coccoides group (**C**), and Clostridium leptum group (**D**). Values are expressed as Mean ± SD, n = 8. Within each panel, values with the superscript symbols “*” and “#” are significantly different from the NC and MC, respectively, *p* at least <0.05.

We also determined the effect of treatments on alteration of gut microbiota community in the mouse model. Compared with that in the NC, gut *Clostridium leptum* group level was significantly decreased in the MC, whereas this decrease was significantly reversed by the treatments (Figure 6D, *p* at least <0.05, except the IMB-L/LAP-L group owing to high variation). Compared with the NC, the MC group had a non-significant decrease of *Clostridium coccoides* group level, whereas the treatments of IMB-L and LAP-H significantly increased *Clostridium coccoides* group levels (Figure 6C, *p* at least < 0.05). Compared with the MC, the treatment of 250 mg/kg of IMB significantly increased levels of *Bifidobacterium* genus, *Bacteroides fragilis* group, and *Clostridium leptum* group (*p* < 0.05). The levels of other gut microbiota groups were not significantly altered by the treatments (Supplement Figure S1). 

### 3.7. Correlation Analysis between Histopathological and Metabolic Parameters

Spearman correlation analysis was applied to determine the association between histopathological parameters and molecular biomarkers in blood or kidney tissues. As shown in Table 3 serum levels of kidney injury markers KIM-1, TIMP-1, cystatin C, lipocalin 2, clusterin, and OPN were significantly positively correlated with all histopathological parameters of kidney lesions (except no significant correlation between interstitial chronic inflammation and KIM-1, or clusterin). Serum levels of cytokines IP-10, VEGF, IL-6, and IFN-γ were significantly positively correlated with all histopathological parameters of kidney lesions. Kidney gene expression levels of inflammatory cytokines IL-6, TNF-α, TGF-β1, and TLR-4 were significant positively correlated with all histopathological parameters of kidney lesions (except no significant correlation between TLR-4 and interstitial chronic inflammation). Kidney gene expression levels of stem cell markers CD133, Pax-2, CD11β, CD44, vimentin, and nestin were also significantly positively correlated with all histopathological parameters of kidney lesions.

The correlation analysis also showed that *Desulfovibrio* genus was negatively correlated with all histopathology inflammation and damage parameters (*p* < 0.05), and that *Prevotella* genus was negatively correlated with tubular dilation and interstitial acute inflammation (*p* < 0.05).

## 4. Discussion

In this study, we evaluated the effects of two novel nutritional components, IMB and LAP, on inhibition of inflammation and kidney injury, and alteration of gut microbiota in the adenine-induced CKD mouse model. With a few exceptions, both IMB and LAP, especially their combinations, significantly inhibited tubular dilation, tubulointerstitial degeneration or atrophy, interstitial chronic inflammation, and acute inflammation in kidneys of the CKD mice. Molecular biomarkers determination showed that IMB or LAP significantly reversed CKD-associated increases of circulating and kidney levels of inflammatory cytokines, circulating levels of kidney injury biomarkers, and kidney levels of stem cell biomarkers. The correlation analysis further indicated significant positive correlations of histopathological markers of kidney lesions to measured molecular markers, with a few exceptions. Gut microbiota analysis also showed that IMB or LAP reversed CKD-associated reduction of *Clostridium leptum* group and *Clostridium coccoides* group; the correlation analysis showed significantly negative correlation between histopathology parameters of kidney lesions and gut *Desulfovibrio* genus or *Prevotella* genus species.

CKD is associated with an increased acute and chronic pro-inflammatory state [3,15]. While inflammation is one of the core pathological features of CKD, it may play a causal role in the development of kidney injury, and contribute to the progression of CKD by inducing release of pro-inflammatory cytokines [15]. The adenine-induced CKD mouse model has been reported to recapitulate several key characteristics of kidney injury and CKD, especially those related to inflammation [12,13], and it has been applied for evaluating the effects of natural products on prevention and treatment of CKD [16,17].

In this study, we found significant reductions in most of the pro-inflammatory markers. The circulating levels of TNF-α, IFN-γ, IL-1, IL-6, and TLR-4 were increased by adenine, but were attenuated by LAP and/or IMB treatment (Figure 3 and Figure 4). Similarly, adenine significantly induced expression levels of MCP-1, IL-1, IL-6, TLR-4, TNF-α, F4/80, TGF-β1, and IL-1β genes in kidney, and most of these increases were attenuated by IMB and/or LAP treatments (Figure 4), which is consistent with the histopathological confirmation of inflammation. Altogether, these findings support that IMB and/or LAP alleviate the kidney injury in CKD at least in part via inhibition of inflammation.

Our results showed that serum levels of TIMP-1, cystatin C, and lipocalin2, markers of kidney injury [18], were high in the adenine-induced CKD mice, but TIMP-1 and cystatin C was significantly reduced by the treatments. The serum level of TIMP-1, which promotes cell proliferation and has anti-apoptotic functions, was increased at the highest level in the adenine-induced CKD mice, and was significantly reduced by these nutritional compounds. To further understand the kidney injury repair mechanism, we determined the gene expression levels of kidney stem cell-related markers. We observed increased expression of key stem cell markers CD44, CD133, Pax-2, CD11β, vimentin, and nestin in the kidney tissues of affected mice, which were all attenuated in the treatment groups (Figure 5). In the human kidney, the expression of CD133 characterizes a population of resident scattered cells with resistance to damage and the ability to proliferate [19,20]. CD133 itself appears to play a functional role in renal tubular repair through maintenance of proliferative responses and the control of senescence [21]. Pax2 [22] is a marker of mature podocytes and is essential for the phenotypic conversion from mesenchymal stem cells to tubular epithelial cells during kidney development. Nestin and CD44 are also involved in cellular proliferation and migration [23,24,25]. Vimentin [26], as a kidney stem cell marker, maintains cells shape and integrity of the cytoplasm, and stabilizes cytoskeletal interactions. WT-1 is now known to have an important role in kidney progenitor cells during development [27]. As kidney injury and kidney stem cell-related markers were increased owing to the CKD, and were significantly or non-significantly reduced by the IMB and/or LAP treatments, the results suggest that, while kidney stem cells related repair is needed in kidney injury, and the effective treatments with IMB and/or LAP reduced the requirement of kidney stem cells for repair.

Microbiota metabolism is emerging as a modifiable risk factor in nephrology, and nutritional manipulation of gut microbiota may play an important role in the prevention and management of CKD [28]. The gut microbiota alterations in CKD was reported by others [29,30,31,32,33], and appear to be the result of metabolic changes. Specifically, CKD patients were reported to have reduced *Bifidobacteriaceae* and *Lactobactria* levels and increased *Bacteroides* levels [34,35]. Interestingly, kidney functions and systemic inflammation were improved by *Lactobactria* treatment in the rat and dog models of CKD [35,36]. Our gut microbiome analysis exhibited alterations in a number of microbial species in the adenine-induced CKD mice model group that were reversed in the treatment groups (Figure 6 and Supplementary Figure S1). However, our data indicate that LAP and IMB do not exert their effects in CKD through altering those bacteria mentioned in other studies. We observed a significant decrease in *Clostridium leptum* levels in the model control mice, which was significantly increased in most of the treatment groups. Some nutritional components such as partially hydrolyzed guar gum could increase *Clostridium leptum* levels and decrease inflammation in a mouse model of colitis [37]. A decrease in *Clostridium leptum* group was also reported in inflammatory bowel diseases and inflamed mucosa of patients with ulcerative colitis [38]. Therefore, more studies in this arena may unravel the link between the decrease in *Clostridium leptum* levels and increased inflammation in CKD. Further correlation analysis also found significantly negative correlation of histopathology inflammation and damage parameters to *Desulfovibrio* genus (Table 3). Inflammatory bowel diseases and inflamed mucosa of patients with ulcerative colitis had a decreased *Desulfovibrio* group level [39]. However, *Desulfovibrio* is also considered as a proinflammatory bacteria [40]. Therefore, the functional role of *Desulfovibrio* in CKD needs further investigation.

## 5. Conclusions

In conclusion, our animal study demonstrated that novel bioactive components, IMB and LAP, significantly inhibited the development and progression of CKD associated with the inhibition of inflammation in kidney tissues and in circulation, improvement of stem cell-based kidney repair, and modulation of gut microbiota. Our results provide essential preclinical evidence to support further investigation on applying IMB and/or LAP for the prevention and alleviation of CKD and associated kidney injury.

## Figures and Tables

**Table 1:** The primer sequences for quantitative real-time (qRT)-PCR. IL, interleukin; TNF, tumor necrosis factor; MCP, monocyte chemotactic protein.

Genes	Forward	Reverse
IL-6	GAGGATACCACTCCCAACAGACC	AAGTGCATCATCGTTGTTCATACA
TNF-α	CATGAGCACAGAAAGCATGATCCG	AAGCAGGAATGAGAAGAGGCTGAG
TGF-β1	TACCATGCCAACTTCTGTCTGGGA	TGTGTTGGTTGTAGAGGGCAAGGA
F4/80	AAGCATCCGAGACACACACAGTCT	TGACTGTACCCACATGGCTGATGA
MCP-1	CTGGTCCGAGTGAGACAAAG	AGATCAGGCTCTGATGGAGAA
TLR-4	CTGCAATGGACAAGGACCA	TCCCACTCCAGGTAAGTG
IL-1β	TAATACGACTCACTATAGGG	ATTTAGGTGACACTATAG
IL-1	CATCCGCAAAGTGGTACGA	AGAAAGACTCCACCAGCCCAGT
CD133	GCCCAAGCTGGAAGAATATG	CAGCAGAAAGCAGACAATCAA
Six2	TCAATGGCAGTGGCAAGTCG	TCAAGCACGGAAAGCAAGCG
Pax-2	TCCCAGTGTCTCATCCATCA	GTTAGAGGCGCTGGAAACAG
Oct-4	CACGAGTGGAAAGCAACTCA	AGATGGTGGTCTGGCTGAAC
CD11b	GACTCAGTGAGCCCCATCAT	AGATCGTCTTGGCAGATGCT
Wnt-4	AGAACTGGAGAAGTGTGGCTGTGACC	TGTATGTGGCTTGAACTGTGCATTCCG
WT-1	ACATCCGACTTCCAAGACAGCACAC	TTGCAGCCAGACCTCTGAAATTCTG
CD29	TGGTCAGCAACGCATATCTGG	GATCCACAAACCGCAACCT
CD44	TCGATTTGAATGTAACCTGCCG	CAGTCCGGGAGATACTGTAGC
Vimentin	CGGCTGCGAGAGAAATTGC	CCACTTTCCGTTCAAGGTCAAG
NANOG	TCTTCCTGGTCCCCACAGTTT	GCAAGAATAGTTCTCGGGATGAA
Nestin	CCCTGAAGTCGAGGAGCTG	CTGCTGCACCTCTAAGCGA

**Table 2:** Statistical Analysis Results of Histopathology Scores in Kidneys.

Histopathology	Grade	NC	MC	IMB-L	IMB-H	LAP-L	LAP-H	IMB-L/LAP-L	IMB-H/LAP-H
Tubular dilation	0	8	0	0	0	0	0	0	2
	1	0	0	0	1	4	1	3	4
	2	0	2	3	3	2	2	4	2
	3	0	6	3	4	2	5	1	0
*p* value (vs. MC)		<0.05				<0.05		<0.05	<0.05
Tubulointerstitium degeneration/atrophy	0	8	0	1	1	0	0	0	2
	1	0	1	1	3	4	0	3	3
	2	0	1	2	3	3	3	3	2
	3	0	6	4	1	1	5	2	1
*p* value (vs. MC)		<0.05			<0.05	<0.05			<0.05
Interstitial chronic inflammation	0	8	0	1	1	3	1	3	3
	1	0	2	1	3	3	5	2	5
	2	0	2	4	3	1	0	3	0
	3	0	4	2	1	1	2	0	0
*p* value (vs. MC)		<0.05				<0.05		<0.05	<0.05
Acute inflammation	0	8	0	0	0	0	0	1	2
	1	0	0	1	3	4	3	3	3
	2	0	2	5	1	1	0	1	2
	3	0	6	2	4	3	5	3	1
*p* value (vs. MC)		<0.05							<0.05

Data were presented as the number of mice that had the damage/inflammation in each group, and were transferred to frequency, followed by the analysis of Kruskal-Wallis test among all groups and then Mann-Whitney test between two groups.

**Table 3:** Significant correlation coefficients between histopathology and hemodynamic parameters, and kidney and gut microbiota gene expression *. KIM, kidney injury molecule; TIMP, tissue inhibitor of metalloproteinases; VEGF, vascular endothelial growth factor; OPN, osteopontin; IFN, interferon; IP, inducible protein.

	Indices	Tubular Dilation		Tubulointerstitial Degeneration/Atrophy		Interstitial Chronic Inflammation		Acute Inflammation	
		R	*p* Value	R	*p* Value	R	*p* Value	R	*p* Value
Coefficient	KIM-1	0.517	0.000	0.341	0.007			0.565	0.000
	TIMP-1	0.708	0.000	0.631	0.000	0.493	0.000	0.759	0.000
	Cystatin C	0.807	0.000	0.811	0.000	0.687	0.000	0.780	0.000
	Lipocalin 2	0.654	0.000	0.606	0.000	0.497	0.000	0.674	0.000
	Clusterin	0.290	0.041	0.242	0.090			0.293	0.039
	OPN	0.659	0.000	0.634	0.000	0.467	0.000	0.735	0.000
	IP-10	0.549	0.000	0.421	0.001	0.227	0.096	0.578	0.000
	VEGF	0.595	0.000	0.541	0.000	0.456	0.001	0.580	0.000
	IL-6	0.461	0.001	0.371	0.011	0.334	0.023	0.532	0.000
	IFN-γ	0.477	0.000	0.398	0.003	0.498	0.000	0.391	0.004
Pathology	Tubular dilation			0.822	0.000	0.768	0.000	0.843	0.000
	Tubulointerstitium degeneration/atrophy	0.822	0.000			0.794	0.000	0.778	0.000
	Interstitial chronic inflammation	0.768	0.000	0.794	0.000			0.697	0.000
	Acute inflammation	0.843	0.000	0.778	0.000	0.697	0.000		
Kidney gene expression	*IL-6*	0.553	0.000	0.420	0.004	0.414	0.004	0.416	0.004
	*TNF-α*	0.386	0.002	0.468	0.000	0.306	0.014	0.365	0.003
	*TGF-β1*	0.370	0.008	0.403	0.003	0.301	0.032	0.351	0.012
	*TLR-4*	0.372	0.030	0.386	0.024			0.410	0.016
	*CD133*	0.456	0.002	0.363	0.015	0.359	0.017	0.512	0.000
	*Pax-2*	0.394	0.002	0.312	0.018	0.342	0.009	0.472	0.000
	*Six2*			0.246	0.073	0.256	0.062	0.231	0.093
	*CD11 β*	0.392	0.002	0.381	0.003	0.315	0.014	0.400	0.002
	*CD44*	0.512	0.000	0.403	0.005	0.486	0.000	0.532	0.000
	*Vimentin*	0.365	0.008	0.325	0.019	0.388	0.005	0.410	0.003
	*Nestin*	0.352	0.005	0.481	0.000	0.351	0.005	0.330	0.009
Gut microbiota	*Desulfovibrio* genus	−0.451	0.002	−0.375	0.012	−0.430	0.004	−0.410	0.006
	*Prevotella* genus	−0.321	0.034					−0.284	0.061

* The data of individual parameter from each animal in all experimental groups were used for correlation analysis.

## Supplementary Materials

The following are available online at https://www.mdpi.com/2072-6643/12/8/2376/s1. Figure S1. Effects of IMB and LAP supplements on gut microbiota levels. Atopobium cluster(A), Clostridium perfringens group (B), Desulfovibrio genus(C), Enterobacteriaceae family (D), Lactobacillus genus (E) and Prevotella genus (D). Values are expressed as MeanSD, *n* = 8. Within each panel, values with the superscript symbols “*” and “#” are significantly different from the NC and MC, respectively, *p* at least <0.05.
